# Discovering Ways That Influence the Older Nurse to Continue Bedside Practice

**DOI:** 10.1155/2011/840120

**Published:** 2011-05-16

**Authors:** LeeAnna Spiva, Patricia Hart, Frank McVay

**Affiliations:** ^1^WellStar Health System, 2000 South Park Place Atlanta, Georgia 30339, USA; ^2^WellStar School of Nursing, Kennesaw State University, 1000 Chastain Road, no. 41 Kennesaw, GA 30144, USA; ^3^WellStar Kennestone Hospital, 677 Church Street, Marietta, GA 30060, USA

## Abstract

A descriptive qualitative approach was used to investigate older nurses practicing bedside nursing and to identify ways to influence older nurses to continue bedside practice. A purposive sample of 18 older nurses was recruited from a healthcare system located in the Southeastern United States. Interpretative analysis of interviews resulted in the identification of three constitutive patterns and eight themes. The first constitutive pattern identified was attributes of the older nurse. The themes comprising this pattern were (a) professional growth in confidence and skills through experience and (b) passion and love for nursing. The second constitutive pattern was enduring stress and frustration. The themes comprising this pattern were (a) physical and mental changes associated with aging, (b) increased patient acuity and patient load, (c) constant change, and (d) time constraints. The third constitutive pattern was enhancements needed for older nurses to continue bedside nursing. The themes comprising this pattern were (a) work environment enhancements and (b) organizational relationship enhancements. Findings may provide a better understanding of the older nurse's role in bedside nursing.

## 1. Introduction

The average age of United States nurses is 46.8 years and approximately 40% of the nursing workforce is over 50 [1, 2]. Furthermore, it is projected that more than 80% of nurses of 40 and older will retire in the next 20 years [3], and the average retirement age is 55 [4]. A critical problem facing nursing and nursing workforce planning is the aging nurse workforce. Older workers are often considered those between ages 55 and 64, but the law defines an older worker as anyone 40 or over. For the purpose of this study, the older nurse was considered to be 55 years or older. 

Nurses retiring at a younger age compared to the general workforce is due to a variety of factors, including financial and family issues, and the heavy physical demands of the profession [5]. In addition, older nurses are more likely to suffer back, neck, and foot injuries and have a reduced capacity to perform physical tasks compared to younger nurses [3, 6–8]. Replacing older nurses with less experienced nurses may be detrimental to patient care quality, patient safety, and an organization's fiscal well-being [9]. Retaining older nurses preserves the important clinical expertise these individuals provide [10]. Older nurses are needed for their skills and expertise [1]. 

The first-ever examination of retention strategies aimed at older nurses was launched by the Robert Wood Johnson Foundation [1]. The project goal was to better understand how to best retain older nurses in the workplace. Researchers surveyed 377 older nurses about what kept them in nursing and administered a demographic questionnaire, 28-item perceptions of fitness for work survey, 27-item perceptions of the work environment survey, 17-item perceptions of human resource practices survey, 9-item likelihood of collegial support survey, and three open-ended questions. Common concerns included heavy patient loads, inappropriate staffing levels, physical demands, emotionally challenging work, inflexible shifts, and feeling unappreciated and undervalued by physicians and leaders. The white paper included an extensive literature review and identified several recruitment and retention strategies but recognized the need for more research on this topic. 

Ward-Smith et al. [11] conducted focus groups with 33 full-time older pediatric nurses at Midwestern pediatric hospital. The researchers found that the nurses had concerns with physical demands, specifically, 12 hours shifts, increased use of computer technology, and mental challenges associated with direct patient care. Suggestions to retain the older nurse at the bedside consisted of assistance with stress management, coping support, benefit flexibility, and help in caring for both children and aging parents. 

In a similar study, Andrews et al. [12] conducted a cross-sectional study and interviewed 84 United Kingdom nurses over 50 years and 18 stakeholders including employers, advisers, and policy makers in nursing. The researchers explored influences on employment related to decision-making for nurses over 50 years of age and investigated the retirement and labor market relationship through older nurses' experience and stakeholders. Semistructured interviews were conducted, and findings highlighted the lack of flexible hours, the stress of work, and lack of benefit flexibility influenced nurses' employment decisions. 

Letvak [7] conducted a qualitative study and interviewed 11 Caucasian female nurses with a mean age of 58 about being an older nurse. Letvak found that older nurses continued bedside practice because they care, carry their load, their relational workplace, and their relationship with the organization. 

Mosley et al. [13] completed an extensive literature review exploring factors that influence the retention and turnover of older nurses. These included the need to respect and recognize the achievements of older nurses, specific managerial characteristics which influence staff retention, the importance of empowerment and autonomy, the valuing of expertise, the provision of challenges, creating a sense of community within an organization, the importance of education and peer development, the impact of work demands and environment, the influence of flexible working and shift options, and the issue of adequate financial reimbursement. 

In an effort to better plan for the aging nurse workforce, it became necessary in our organization to gain knowledge of older nurses' experiences of practicing bedside nursing. Our organization is faced with an aging nurse workforce, and there was a need to identify ways to influence older nurses to continue practicing bedside nursing. Therefore, we conducted a research study with the following objectives: (a) to describe the experience of the older nurse practicing at the bedside and (b) identify ways to retain the older nurse at the bedside. This paper describes older nurses' experiences of practicing bedside nursing and actions taken by our organization to influence older nurses to continue bedside practice.

## 2. Design

A descriptive qualitative approach explored older nurses' experience of bedside practice as conceptualized and grounded in personal experience.

## 3. Methods

### 3.1. Sample and Setting

A purposive sampling method was used in an effort to include a diverse group of participants. Inclusion criteria were (a) registered nurse in direct patient care, (b) 55 years of age or older, (c) able to speak and read English, and (d) willing to participate in an audiotape interview. This study was conducted between April 2010 and July 2010 in an integrated healthcare system located in a Southeastern state. The integrated healthcare system consists of five hospitals, physician practice groups, and outpatient services. Research participants were limited to older nurses practicing at the bedside and were recruited through notices in the facilities, nurse leaders, referrals by potential research participants, colleagues, and acquaintances.

### 3.2. Data Collection Procedure

Researchers typically have a personal history with what they choose to study; otherwise, they would never have considered it worth pursuing [11]. Arising from one's personal history with the phenomenon of interest will be assumptions that have been made about the phenomenon. Heidegger asserted that it is not possible to bracket our assumptions about the world but that we can become aware of our assumptions and attempt to keep them at arm's length. Before beginning data collection, the researchers discussed their assumptions for this study. 

Ethics approval was obtained from the Nursing Research Committee and Institutional Review Board for the Protection of Human Subjects. The initial contact with the nurses was either face-to-face or by telephone conversation. Written informed consent was obtained from each participant at the beginning of the interview. Interviews were conducted at either the facility or participant's home and interviews lasted from 30 minutes to 1 hour and 30 minutes. In order to protect confidentiality, the researcher assigned each participant a pseudonym. The pseudonym was used when describing and discussing the study findings. Only the researchers had access to the list of participants' names and pseudonyms. Due to the tape-recorded interviews, anonymity was not possible. Audiotapes were transcribed by a transcriptionist from whom the identities were withheld. 

The interviews were semistructured using an interview guide as recommended by Patton [14] and Lincoln and Guba [15] to assure that each question was discussed. The participant was asked to describe his or her experience of bedside nursing and to identify ways that influence the older nurse to continue bedside practice in as much detail as he or she can recall. The researchers continued interviewing older nurses until repetition of the themes was achieved. The researchers recognized the repetition and determined that the addition of new participants confirmed the findings rather than added new information. The repetitive nature of data indicated that saturation was achieved.

 In addition to the tape-recorded interviews, a reflexive journal was kept by the researchers [15] that contained the record of insights, recurring words or phrases, ideas, thoughts, questions, concerns, and decisions made during the research process. The notes included unrecordable events during the interview, such as context, participant affect, and body language. This served as a personal diary.

### 3.3. Data Management/Analysis

The constant comparative method of data analysis was used [15]. The constant comparative analysis in this study proceeded in the stages outlined by Diekelmann and Allen [16] and extended by Minick (P. Minick, personal communication, April 11, 2003). 

Verbatim transcripts of the interviews served as the data for analysis. A computer software program, *Microsoft Word 2007*, was used to number each line of the texts and later to sort and manage the data. Data analysis was accomplished by using a research team composed of the researchers and another nurse researcher. This team approach fostered insight and enhanced methodological rigor of the study. 

Each member of the research team prepared a written summary of each interview to gain an overall understanding of the interview. The summary included key words, phrases, and paragraphs which best represented the participant's message. The purpose of this analysis at this point was to get a feel for the overall meaning of the participant's stories. The research team met and each member read his or her interpretation aloud. The interpretations were discussed in depth, and points of congruence and difference were identified. When interpretations were different, the research team explored the possible sources of the differences and returned to the text to come to a level of consensus. Data collection and this initial analysis occurred concurrently. When the initial analysis of the text was completed, the researchers used *Microsoft Word 2007*, to code each section of the interview using the participants own words to label the data, so we stayed close to the participants words rather than being conceptualized by the research team. A code book was developed listing each code and the initial definition of the code to maintain consistency in labeling. Once coding was completed for the individual text, then all data within each code was read and reread individually. Codes containing similar data were collapsed into categories. Categories were labeled with words from participants as close as possible. Similar categories were collapsed into the major themes found in the data. The researchers printed hard copies of coded segments to examine them more closely for common meanings, returning to the initial analysis summaries of the interviews, and rereading the researcher's notes made at the research team meetings. This strategy identified patterns that were found in the interviews. The entire analysis was reviewed by the research team, as well as, by another researcher who is familiar with the research method. The purpose of this process, or review, is to validate the authenticity of the analysis. Revisions were made to clarify and strengthen the labels. The research participant was the final authority of the interpretation. The participants critique was sought to determine the validity of the interpretation. The researchers asked participants to read and respond to the entire interpretation. Their responses and suggestions were incorporated into the final draft.

Lincoln and Guba [15] presented four criteria (credibility, dependability, confirmability, and transferability) for achieving trustworthiness in qualitative research. Credibility in this study was addressed through the use of a research team, member checks, reflexive journal, and audit trail. To address the credibility of the interpretations of the data, experienced qualitative researchers from the research team interpreted transcripts. The circular hermeneutic method [16] enhanced credibility, as the data were returned to repeatedly by the research team. Regular meetings were scheduled with the research team to assure that interpretations were grounded in data, giving expert consensual validation. 

“Member checks” were made with the participants, to discuss the interpretations of their stories and the larger conclusions regarding themes and patterns [15]. An “audit trail” was kept, consisting of a reflexive journal, audiotapes, and transcripts of the interviews, notes, and computer data [15]. 

Transferability was addressed through a reflexive journal that provided a record of contextual data, including descriptions of the settings and decisions that affect the data. The audit trail described earlier enabled the reader to evaluate the context.

## 4. Results

### 4.1. Sample

The sample consisted of 18 older nurses working in a healthcare system located in the Southeastern United States, 17 female and one male. Nurses ranged in age from 55 to 67 years (*M* = 61, SD = 3.7). A majority of the nurses were Caucasian (*N* = 14), with the next largest groups being African American (*N* = 2) and Asian (*N* = 2). A majority held baccalaureate degrees (*N* = 10), two held master's degree, three held diploma, and three held associate degrees. A majority rated both their current health (*N* = 13) and financial status (*N* = 14) as good. 

A majority had been a nurse for 26–30 years (*N* = 13), worked fulltime (*N* = 16), and worked on a medical surgical unit (*N* = 9). Four nurses plan to retire in two years, nine plan to retire in three to six years, and nine plan to stay in their current role but reduce their hours before retiring.

### 4.2. Interpretative Analysis

Interpretative analysis of interviews with 18 older nurses resulted in the identification of three constitutive patterns and eight themes. Patterns identified in every interview were considered constitutive patterns. Common experiences were grouped together and identified as themes. The first constitutive pattern identified was attributes of the older nurse. The themes comprising this pattern were (a) professional growth in confidence and skills through experience and (b) passion and love for nursing. The second constitutive pattern was enduring stress and frustration. The themes comprising this pattern were (a) physical and mental changes associated with aging, (b) increased patient acuity and patient load, (c) constant change, and (d) time constraints. The third constitutive pattern was enhancements needed for older nurses to continue bedside nursing. The themes comprising this pattern were (a) work environment enhancements and (b) organizational relationship enhancements.

### 4.3. Attributes of the Older Nurse

The first constitutive pattern that emerged was attributes of the older nurse. All nurses told stories about their experience and how their knowledge and confidence had grown over the years. Stories were told about their passion and love for nursing and being able to effectively interact with patients and families.

#### 4.3.1. Professional Growth in Confidence and Skills through Experiences

 All nurses except for one nurse with three years of nursing experience spoke about how they have grown as a nurse over the years through life experiences. Attributes that described the nurse included having a connection to patients, familiarity to various patients and disease processes, organizational skills, preparedness, calmness, dependability, knowledge, confidence, and a strong work ethic. Each nurse expressed how they had acquired these skills through life experiences. 

Nurses felt a connection to their patients either because they had experienced a similar disease process or dealt with a similar life experience. One nurse illustrated this point, “There are many benefits because you are an older nurse. There are a lot of life history happenings and you can relate more to patient issues.” One nurse had a history of cancer and worked on an oncology unit and stated, “I am a cancer survivor and I can relate to my patients.”

The nurses felt prepared, organized, and knowledgeable to deal with any situation. One nurse stated “I take care of routine tasks so I am always prepared for emergencies.” Another nurse expressed “I have learned over the years to be organized and remain calm.” Nurses had a sense of security by the knowledge and experience obtained over the years. “Having knowledge, confidence, and experience are rewarding and you have a sense of security.”

Several expressed they learned from their patients. “We have a different way of looking at things. I can look into a patient's eyes and see and know how they feel.” Several felt that they were calmer and worked better with patients and families. A nurse of 30 years responded

I do not get ruffled and nothing really shocks me. I am able to deal better with situations, different personalities, and difficult patients. It feels good to have the knowledge and experience in which I did not have years ago.

Seven nurses discussed intergenerational conflicts with younger nurses. One nurse stated “Younger nurses need to respect and listen to the older nurses due to our years of experience. The younger nurses sometimes have a hard time doing this.” Another nurse felt that the work ethic had changed “I see the younger nurses and if they do not feel good, they take the day off. Maybe this is a good thing and they will live longer and not have as much stress. I take my job very seriously.” Several nurses felt they had a better connection with their patients compared to younger nurses. One nurse stated “I can relate to patients better. I can talk to patients and family easier than younger nurses.” Another nurse felt “Younger nurses have difficulty respecting our experience and age.” A mother and baby nurse of more than 30 years stated “I have trouble tolerating the younger energetic nurses; however they have fresh ideas.”

#### 4.3.2. Passion and Love for Nursing

 The passion and love for nursing was exhibited throughout the interviews. One nurse expressed “Over the years, I have become frustrated but have never regretted choosing nursing as a profession. It continues to be the best thing I could ever imagine doing. I could not imagine doing anything else.” Several felt that nursing was a calling. “I love working so much, feel like it is a gift from God. I love bedside nursing, it is a gift.” Another nurse stated “I was born to be a nurse.” The data reveals that the nurses felt both professional and personal gratification from their role as a nurse.

### 4.4. Enduring Stress and Frustration

Every nurse told stories about being frustrated with the physical work demands and other unexpected physical and psychological outcomes that altered their role as a nurse. The nurses found their experiences to be both stressful and frustrating, yet they persevered in spite of the difficulties. Frustration and concerns about patient acuity and load, technological changes, policies, and paperwork were expressed. Several spoke about having limited time to spend with their patients due to these constraints.

#### 4.4.1. Physical and Mental Changes Associated with Aging

 Several nurses discussed the loss of endurance which may accompany aging and illness and how they have learned to live with these outcomes. Nurses attributed their loss of energy to aging processes or to other illnesses. For all these nurses, their loss of energy meant changes in how they lived their daily lives. Many described bedside nursing as physically exhausting resulting in a lack of energy and not being able to maintain the fast pace. One nurse illustrated this point “Each year it is harder physically, mentally, and it makes me wonder if this is what I want to continue doing; however I would miss my friends, patients, and families and feeling productive.” Another stated “The role is very demanding, physically, emotionally, spiritually, and mentally taxes you on every level. I have had to make changes over the years to accommodate.” Several of the older nurses either transferred to less physically demanding positions or decreased their work hours to accommodate for their physical limitations. Several nurses were worried about aging limiting their ability to continue bedside nursing. While some nurses experienced physical discomfort, others reported mental changes. As one nurse expressed “The downside is memory changes and being on top of things has deteriorated.” Another stated “I do not have the mental stability that I had.” These declines in memory may have resulted from any one or a combination of factors such as aging processes or other disease states. Another interpretation is that they understood how vulnerable they were and that was frightening and stressful. 

The theme dealing with physical and mental changes associated with aging included learning to live with energy loss and dealing with deficits. These data indicated that physical demands of the role have a significant impact on older nurses. At times, it was hard to distinguish whether nurses were experiencing changes because of normal aging processes, or if changes were signaling a disease state. In spite of the stress and frustration they described, they found ways to live with unexpected outcomes by either finding new ways to deal with the outcome or accepting the outcome. Many were worried about aging limiting their ability to continue bedside nursing.

#### 4.4.2. Increased Patient Acuity and Patient Load

 The most frustrating experiences for nurses were when they could not provide good care to their patients because of high acuity patients and heavy work load. One nurse expressed

We are now doing surgeries on heavier and sicker patients. It is harder for the aging nurse to physically take care of these patients. Most patients are not young enough to participate in self-care. It is frustrating that we are asked to take care of patients three times our size.

Many nurses felt that staffing should be based on acuity versus number of patients. In addition, thought should be placed into patient assignments and the fast turnover of patients should be accounted for. One nurse articulated this point “Not a lot of thought is placed into the nurse's assignments and the fast flow of patients in and out is not accounted for.”

Areas of frustration mentioned by older nurses were lack of staffing and inconsistent staff. Nurses felt that their workload was heavy and they were doing more with less. One medical-surgical nurse stated “Caring for six patients makes a busy day and you're on your feet the whole time except for when you have a break. It gets tiring and sometime becomes an obstacle.” These points illustrated that nurses are caring for sicker, older, and more diverse patients with more chronic conditions at the bedside.

#### 4.4.3. Constant Change

 Several nurses discussed aspects of constant change that caused frustration and stress. The electronic medication administration record (MAR) and electronic documentation system were constantly changing. As one nurse commented “Many of the nurses do not like not having the paper MAR. I like to look and see when medications were given last and when needed again- you have to look everything up in the computer.” This change involved learning new skills related to technology.

Several nurses felt constant changes made it difficult to maintain job duties and provide acceptable patient care. One nurse illustrated this point

We are in constant change. Paperwork and policies change constantly. All of the changes make it hard to maintain our responsibilities and continue to provide excellent patient care.

Another commented “Older nurses are not as receptive to accept change. It is part of growth and adapting and has become more accepting in the last 10 years. It is a challenge to learn new technology and watch medicine advance.”

#### 4.4.4. Time Constraints

 Another source of frustration arose when nurses did not have time to complete their work and less time to devote to their patients. Nurses spent a majority of their time completing paperwork and calling physicians. One nurse stated “There is not enough time to finish your day. I spend all of my time communicating between doctors. I had rather have time to devote to my patients versus paperwork.” One nurse felt that she did not have time to really know what was going on with her patients and illustrated the importance of time “When you have time, reading the chart, knowing what the doctor is saying, and knowing the whole picture is critical to patient care.”

Another stressor affecting nurses came as each had to assume new responsibilities. One described not having time to orient nurses and the stress this caused. “If you are assigned to a new graduate nurse, you do not have time to devote to the new graduate and patients suffer and the aging nurse is a wreck.” 

### 4.5. Enhancements Needed to Continue Bedside Nursing

The third constitutive pattern was enhancements needed for older nurses to continue bedside nursing. Nurses provided several ideas to enhance their practice including work environment, human resource, and leadership enhancements.

#### 4.5.1. Work Environment Enhancements

 Work environment enhancements included the unit's physical layout, technology and equipment, staffing, support and teamwork, and educational enhancements. Many spoke of work redesign ideas that would make it easier to continue bedside practice. These included eliminating semiprivate rooms, providing more workspace to decrease standing, and having more ergonomically friendly equipment to assist with turning and lifting patients. Ideas to assist with decreasing walking included having supplies, computers, locked medication drawers, and equipment located at the bedside; making patient assignments closer together; and having a circle layout nursing station design versus the long hallways. One nurse illustrated this point “Depending on the layout of the floor, it can be physically challenging depending on location of the medication room, walking, and pushing around computers.” One nurse felt isolated due to the nursing unit design. “You're isolated and do not always know who your working with due to the geographical make up of the unit and having patients in a central location would avoid less walking.” A majority of nurses expressed the need to eliminate the semiprivate rooms, “The semiprivate rooms are small and you have to pull furniture and beds out to accommodate for another patient.” Several nurses expressed the need for more workspace. “More workspace at the nurses' station is needed. “A lot of times you have to stand to chart and sometimes this is the only time you can sit down.”

All nurses spoke about the technological and equipment enhancements that were needed to continue bedside practice. The increased demand of documentation was overwhelming, and the computers slowed their workflow down significantly. Many felt they were duplicating their efforts. One nurse illustrated this point

Duplicating efforts in both computer and paper this impedes my work. The same questions are asked on several forms and it takes a lot of time away from the bedside. We need to take advantage of the technology and not have hard copies of paperwork.

All nurses discussed the need to care for fewer patients so they could provide better care. 

 “The older nurse is slower due to age and having a lower patient to nurse ratio would help so you know what is occurring with the patient.” Several nurses expressed the importance of teamwork and how this made their role easier. One nurse stated “Nursing techs do a lot of lifting and turning and this is a challenge for me.” Nurses felt teamwork could be enhanced between each other “Other nurses not being proactive and leaving a lot for the next shift.”

#### 4.5.2. Organizational Relationship Enhancements

 Organizational relationship enhancements targeted human resource and leadership enhancements. Human resource enhancements included positions that require less physical demands, scheduling, and benefit enhancements. Several nurses have transitioned to less physical demanding roles. “Several years ago, I had to transfer from an intensive care unit to a less physically demanding role.” Another commented “I have taken advantage of working in areas with less physical and mental requirements.” Several of the nurses described less physical demanding positions.

Positions need to be developed for less physical demanding roles such as teaching, mentoring, committee work, completing audits and paperwork, and admission-discharge role.

Almost all nurses expressed concerns with the work hours. The 12-hour shifts were physically demanding for nurses. Many had either decreased to part time or if allowed, decreased to eight hour shifts. Several had no schedule flexibility evidenced by one nurse “We are better suited for 8-hour shifts. There is a lack of part-time positions.”

Several nurses suggested benefit enhancements such as decreasing work hours and working a shorter workweek but maintaining the same benefits. One nurse commented “Employers do not offer anything like teaching offers- after 10 years be offered to take off one year and return to your bedside position.” Several talked about their salaries. The lack of pay increase with experience “I only make $15,000 more than a new graduate nurse. Experience should compensate for something.” 

Leadership enhancements included less budget focus and developing a closer connection to the bedside nurse. Many felt a disconnection between the bedside nurse and leadership. One nurse commented “Administration is to far removed and has no idea. Administrators care about business and dollars. Decisions administrators make without knowing what it is like to take care patients is unfair.” Another nurse commented “Administrators have never come down to see what needs to be done in order to meet unrealistic goals that they are making. I do not feel the organization values the older nurse; they would let us go at a drop of a hat because we are expensive.”

Nurses felt managers were more budget focused than staff focused. “Managers are under the gun regarding budget.” Some felt that management was not supportive. “Management is afraid to speak up. I do not feel supported. We express our concerns but the concerns go nowhere.” Several nurses expressed different views of leadership as one nurse stated

The upper management seems like they are trying, however, there is still resistance keeping the momentum. They are more willing to look at issues to make the process better than middle management…. Historically, I have found if you bring a problem to management rather resolve the issue, they add another tool.

Another nurse stated

The fact that you are performing these interviews it appears to me that upper management is looking at what the older nurse can provide….Over the past years, I felt like administration wanted to get rid of the older nurses. Now they are more open to realize experience is good.

## 5. Discussion

The study findings expand and deepen understanding of the experiences for older nurses practicing at the bedside and identify ways to influence older nurses to remain practicing at the bedside. The description of the older nurse experience brought themes together in a way that is distinct from other descriptions of the older nurse's experience in the research literature. Older nurses expressed concerns about being able to provide quality care due to higher patient to nurse ratios and higher acuity patients being placed on medical surgical units. Inadequate staffing plans and inconsistent staffing patterns added to their frustration. Frustration was expressed with not having time to complete their work or spend time with their patients. They expressed concerns with having to spend more time with charting, organizing care between physicians, and eliminating roadblocks in care processes.

Consistent with the research findings in other qualitative studies of older nurses, the most pervasive and difficult experiences in the sample of older nurses involved dealing with stress, frustration, constant change, physical and mental declines, and dealing with intergenerational conflict [7, 17–19]. Respondents vividly described their physical and mental declines that they struggled with daily evidenced by the following statement “Daily, I struggle with both physical and mental limitations due to aging.” Older nurses also detailed how much their lives had changed by choosing nursing as a profession evidenced by the following quote “Nursing has provided me opportunities to learn and grow, provided me a different way of looking at life, and prepared me to deal and survive cancer.” 

Yet despite the physical and mental declines, stress, and frustration, most found positive aspects of and meaning in being a nurse. Meaningful and joyous times were created through the recollection of earlier and happier memories, making a connection with patients, and cherishing the preciousness of caring for patients and families. 

This study provided insight into how to implement strategies to keep older nurses practicing at the bedside as supported in the literature [1, 20]. These ideas expressed in their narratives included suggestions related to work environment and organizational enhancements. Work environment enhancements included the unit's physical layout, technology, staffing, and support and teamwork opportunities. Staffing enhancements included consistent patient to nurse ratios, having an admission and discharge nurse, and patient assignments based on acuity. Work redesign ideas included eliminating semiprivate rooms, providing more workspace to decrease standing, locked medication drawers in patients' rooms, storing supplies and equipment within patients' rooms, bedside computers, user friendly computer systems, and nursing units designed within a circle layout to reduce walking. Supported in previous research, older nurses suggested organizational enhancements including positions that required less physical demands [1, 21], flexible scheduling, and benefit options [22, 23].

Older nurses expressed a desire for leadership to have a better understanding of their role [7, 17]. Being recognized for their expertise, respected, and acknowledged for a job well done were important to older nurses [18, 23, 24]. Promoting an organizational culture that values the knowledge, experience, and skills of the older nurse is essential in building an environment that fosters the satisfaction and retention of older nurses [13, 22].

## 6. Practice Implications

Instead of only identifying strategies to retain older nurses as previous researchers have done, our organization implemented several of their recommendations. Our organization is building a new hospital and new patient tower, and older nurses are assisting with nursing unit designs. With expansion of a new patient tower, semiprivate rooms will be eliminated by May 2012. One hospital in the organization developed an admission and discharge nurse team. The average nurse on this team is 50 years of age. The organization is revamping the preceptor program and planning to incorporate views and suggestions from older nurses. By July 2011, the organization is planning to lower the nurse patient ratio in all medical surgical and telemetry nursing units. During nurse week, an older nurse luncheon was scheduled to recognize older nurses for their contributions to patient care. In March 2011, the organization went live with a redesigned and more efficient electronic medical record that incorporated a majority of paper forms into an electronic format. Also, information technology (IT) is working on deploying an in-room computer device in every patient room.

## 7. Limitations

A limitation of the study was that the interviews were conducted in a homogenous sample of Caucasian older female nurses that had 26 to 30 years of experience as a nurse in one integrated healthcare system in the United States. Only one male was represented within the sample. Male and female nurses may potentially have different and unique views and issues related to working as older nurses at the bedside. Additionally, cultural differences and years of experience may impact how nurses perceive their work environment resulting in varying needs and issues. Findings of the study would be strengthened by replicating the study in a more ethnically diverse nurse sample with representation of the male population, in samples of nurses in diverse geographic areas, and older nurses entering nursing as a second career to determine if similar findings exist.

## 8. Conclusion

Hearing the voices of older nurses painted a colorful picture of how they interpreted and envisioned practicing as a bedside nurse allowing others to view the canvas and learn from their experiences. Valuable lessons and creative ideas can be learned by listening to older nurses and addressing important issues identified by this valuable aging nurse workforce. As in this study, strategies can be implemented to encourage and retain older nurses at the bedside. Older nurses provide wisdom, knowledge, and strength that construct a foundation for the nursing profession, particularly, as a competent bedside practicing nurse.
